# Effects of suicide on psychiatry trainees

**DOI:** 10.1192/bjb.2019.46

**Published:** 2019-08

**Authors:** Marilia Calcia, Alice Debelle

**Affiliations:** Consultant Liaison Psychiatrist, South London and Maudsley NHS Foundation Trust, UK. email: marilia.calcia@nhs.net; Specialist Registrar in Child and Adolescent Psychiatry, South London and Maudsley NHS Foundation Trust, UK

We read with interest the survey by Gibbons *et al*^1^ and welcomed the idea of examining what kind of support may help clinicians deal with this difficult aspect of mental health practice.

It is interesting to note how some responses to the survey indicated that having experienced a patient suicide as a trainee had a significant influence on the responder's choice of subspecialty. This finding highlights how junior doctors, whether in a formal training programme or not, are particularly vulnerable to the adverse effects of a patient suicide, with potential effects on recruitment and retainment in psychiatry.

In our mental health trust in South East London, trainee-led initiatives in collaboration with the postgraduate training department have been making changes to the learning and support offered to junior doctors who are involved in a serious incident investigation in the past 5 years.^2^ The projects involve annual events to promote knowledge and discussion about quality and safety; discussions about the process of serious incident investigations and the support available at each junior doctor induction; and the development of a written resource on formal and informal sources of support for junior doctors involved in a serious incident investigation. These projects are under constant review in order to respond to trainee feedback.

We thank the authors for the valuable work exploring how suicide can affect doctors of any grade. We hope that mental health trusts and postgraduate training departments continue working to develop formal and informal support structures for doctors experiencing this difficult event.
